# Guided Bone Regeneration (GBR) for Reconstructing Alveolar Bone Defects Following Resective Treatment of Medication‐Related Osteonecrosis of the Jaws (MRONJ) Lesions: A Case Series

**DOI:** 10.1002/ccr3.71404

**Published:** 2025-11-12

**Authors:** Daya Masri, Omar Ghanaiem, Vadim Raiser, Shaked Adut, Gavriel Chaushu

**Affiliations:** ^1^ Department of Oral and Maxillofacial Surgery, The Maurice and Gabriela Goldschleger School of Dental Medicine Tel Aviv University Tel Aviv Israel; ^2^ Department of Oral and Maxillofacial Surgery Rabin Medical Center Petah Tikva Israel

**Keywords:** bone augmentation, guided bone regeneration (GBR), medication‐related osteonecrosis of the jaws (MRONJ), reconstruction, resection

## Abstract

This case series describes three patients with medication‐related osteonecrosis of the jaw (MRONJ) treated by surgical resection combined with guided bone regeneration (GBR). All patients had a history of prolonged antiresorptive therapy and developed stage 3 MRONJ following dentoalveolar procedures. Treatment consisted of marginal resection or sequestrectomy followed by GBR using combinations of different bone substitutes, membranes, and recombinant human bone morphogenetic protein‐2 (rhBMP‐2). Favorable healing was observed in all cases, with progressive bone regeneration confirmed radiographically and restoration of oral function achieved in two patients through successful implant rehabilitation. These findings suggest that MRONJ resection combined with GBR offers an effective, less invasive alternative to extensive reconstructive procedures in selected MRONJ patients, supporting both functional and esthetic rehabilitation while minimizing morbidity.

## Graphical abstract

This composite figure illustrates some of the clinical and radiographic features of medication‐related osteonecrosis of the jaw (MRONJ), including panoramic, intraoral, axial CBCT, and extraoral images highlighting intra‐oral to bone probing, radiographic lysis, sequestration and periosteal reaction, and facial swelling.

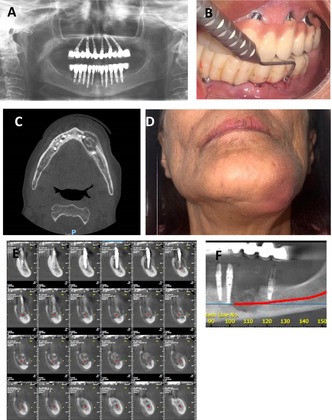

**Summary**

- Surgical resection of MRONJ lesion combined with guided bone regeneration offers a viable, less invasive alternative for treating MRONJ, promoting bone healing and functional rehabilitation while minimizing the need for extensive reconstructive procedures.

## 1 Introduction

Medication‐related osteonecrosis of the jaw (MRONJ) is a severe adverse drug sequelae characterized by progressive bone destruction in the maxillofacial region. It occurs predominantly in patients receiving antiresorptive drugs, such as bisphosphonates and denosumab, or antiangiogenic treatment [1, 2, 3]. The American Association of Oral and Maxillofacial Surgeons (AAOMS) defines MRONJ as exposed bone, or bone that can be probed through an intraoral or extraoral fistula, persisting for more than 8 weeks in patients with a history of antiresorptive or antiangiogenic agent use, without prior radiation therapy to the craniofacial region [4]. Epidemiologically, MRONJ remains relatively rare but poses significant morbidity. The incidence varies based on the underlying condition and medication regimen, with higher rates observed in cancer patients receiving high‐dose intravenous bisphosphonates (< 5%) compared to those treated for osteoporosis (< 0.05%) [4].

Clinically, MRONJ presents with pain, swelling, infection, exposed necrotic bone, sensory nerve deficit, pathological fractures, and sinusitis leading to impaired quality of life [4, 5, 6]. The condition can cause difficulties in mastication, speech, and social interactions, imposing a considerable health burden on affected individuals [7].

The pathophysiology of MRONJ is complex and multifactorial and involves genetic predisposition, systemic underlying conditions, cumulative dose and duration of antiresorptive therapy and local factors such as inflammation, surgical procedures, and tooth or implant extraction [8, 9]. Dentoalveolar surgery is often recognized as a local risk factor for the development of MRONJ [4]. For instance, tooth extraction is reported as a predisposing factor in approximately 62%–82% of MRONJ cases [10, 11]. Therefore, a significant hesitancy is shown among clinicians to pursue surgical intervention in MRONJ patients.

On the other hand, surgical intervention is increasingly recognized as a highly effective approach for treating MRONJ lesions, demonstrating high success rates across all disease stages [4]. Segmental or marginal mandibulectomy and partial maxillectomy have proven effective in managing MRONJ, including early‐stage cases. These procedures involve removing the infected bone beyond the necrotic margins to reach healthy, bleeding tissue [12, 13, 14]. According to the above scientific evidence, surgical intervention appears to play a dual role in MRONJ—both potentially triggering and promoting the healing of lesions—thereby raising important questions about its classification as a risk factor.

Despite the potential benefits of surgical management, clinicians tend to avoid surgical interventions in MRONJ patients. This reluctance stems from concerns that surgical procedures, particularly dentoalveolar surgeries, may complicate the condition or trigger new lesions [4]. However, evidence shows that surgical treatment will likely yield better outcomes compared to nonsurgical management. For instance, a study comparing treatment modalities in stage II MRONJ found that surgical intervention led to a higher success rate (89.3%) compared to nonsurgical treatment (33.3%) [15]. These findings highlight the need to reassess the role of surgery in MRONJ management, balancing potential risks against the benefits of definitive treatment.

Severe maxillo‐mandibular defects might need to be reconstructed using free vascularized bone or using reconstruction plates. While a titanium plate can provide temporary stabilization [16], failure to reconstruct with bone increases the risk of soft tissue contraction, plate exposure, and infection, potentially requiring additional surgery [17]. However, these procedures have a high morbidity including donor site complications, lengthy operating time, and intensive postoperative care required. Furthermore, the inherent complexity of the procedure, combined with the often‐compromised health status of elderly MRONJ patients, may increase procedural morbidity and adversely affect its success rates [18].

In response to the challenges associated with extensive surgical procedures in MRONJ patients—and considering the ongoing controversy surrounding surgical intervention, being both a potential risk factor and an effective therapeutic approach, our Maxillofacial Surgery Department of Rabin Medical Center has implemented a treatment approach combining surgical resection with GBR for managing MRONJ lesions, aiming primarily to minimize the need for extensive or major surgical procedures in affected patients. This approach involves debridement of necrotic bone with the application of bone augmentation materials, aiming to preserve as much healthy tissue as possible and eliminate the need for more extensive and invasive procedures.

Through this case series, we present three patients diagnosed with MRONJ, varying in age and underlying conditions. These patients were managed using our standardized protocol, with careful monitoring during the postoperative period.

## 2 Case Presentation

### 2.1 Case 1

#### 2.1.1 Case History and Examination

**FIGURE 1:**
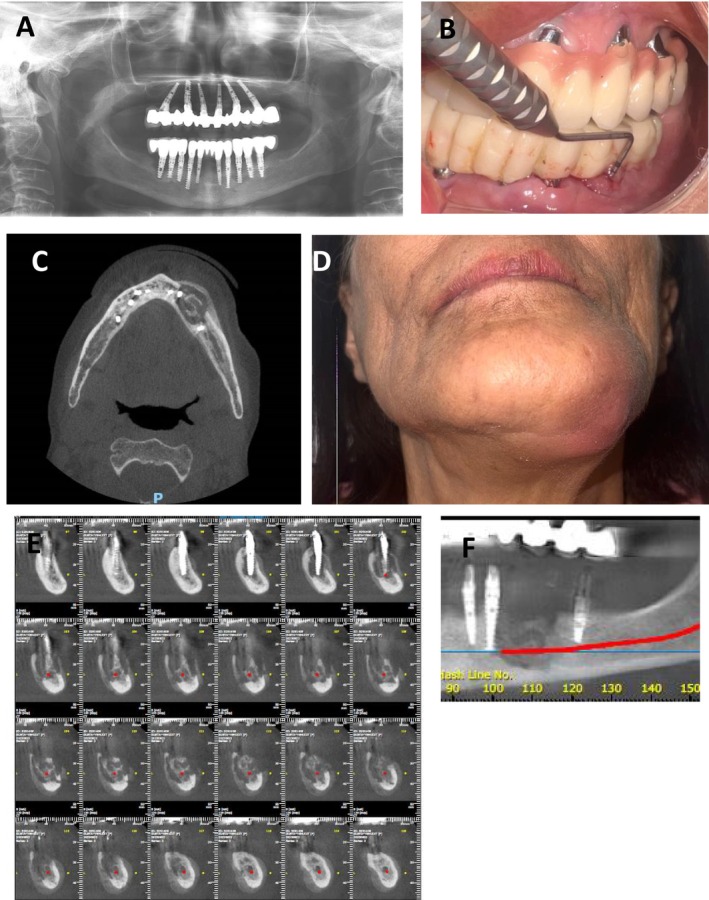
Case history and examination—Case 1. (A) A panoramic radiograph demonstrating the mandibular region prior to dental implant removal and MRONJ development. (B) Intraoral clinical image showing probed bone with purulent discharge through a fistula in the left posterior mandible. (C) Axial CBCT view obtained at the initial presentation, revealing areas of osteolysis and bony sequestration in the left mandibular body. (D) Extraoral photograph illustrating facial swelling on the left side of the mandible during the initial clinical evaluation. (E, F) Additional CBCT slices from the first visit further confirming the extent of bone destruction and sequestration in the affected region.

A 69‐year‐old female patient (G.Y.) presented with acute pain, swelling, redness, and paresthesia localized to the left mandible. Intraoral examination revealed an intraoral fistula, purulent discharge, and exposed necrotic bone in the posterior mandible, raising immediate clinical concern for MRONJ. Her medical history was notable for Hypercholesterolemia managed by Rosuvastatin and osteoporosis, managed with vitamin D, calcium supplements and oral bisphosphonate (Risedronate) for over 7 years. Notably, 2 months prior to presentation, she underwent the removal of two dental implants from the same region without a preoperative drug holiday. A cone‐beam computed tomography (CBCT) scan obtained at the time of evaluation revealed an extensive osteolytic lesion involving the left mandibular body, consistent with MRONJ stage 3 as defined by the AAOMS [4] (Figure 1).

#### 2.1.2 Methods (Differential Diagnosis, Investigation, and Treatment)

**FIGURE 2:**
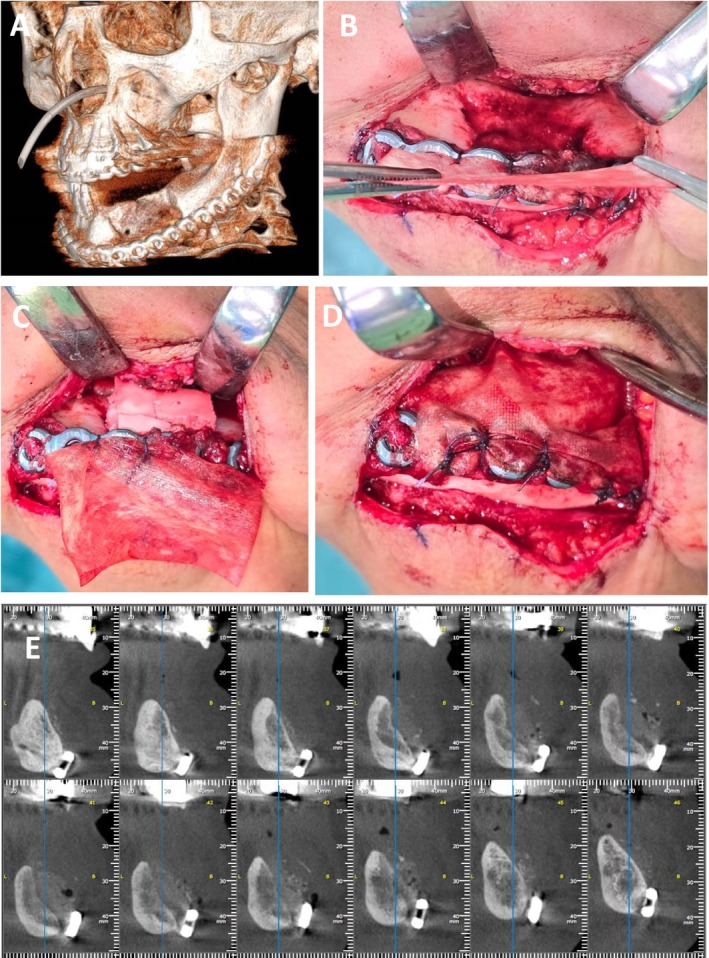
Bone augmentation 9 months following marginal mandibulectomy—Case 1. (A) Three‐dimensional reconstruction from multidetector computed tomography (MDCT) immediately following marginal mandibulectomy and fixation of a reconstruction plate, demonstrating the extent of resection and stabilization. (B) Submandibular surgical approach revealing the reconstruction plate and the bone defect, OSSIX Agile membrane is sutured to the reconstruction plate. (C) The mandibular defect augmented using an autologous bone graft harvested from the anterior iliac crest, in combination with OSSIX Bone. (D) OSSIX Agile membrane covering the bone graft. (E) Eighteen‐month follow‐up Axial CBCT.

Initial management included a course of systemic antibiotic therapy and antimicrobial mouth rinses, along with a temporary drug holiday from Risedronate. A biopsy of the exposed bone was performed, and the diagnosis of MRONJ was confirmed through histopathological examination. Following confirmation, the patient underwent a marginal mandibulectomy under general anesthesia, during which the failing implants were extracted and the necrotic bone was resected reaching healthy margins, and a reconstruction plate was placed to stabilize the mandible (Figure 2A).

At the 9‐month follow‐up, a second surgical procedure was performed under general anesthesia. The mandibular defect was augmented using an autologous bone graft harvested from the anterior iliac crest, in combination with OSSIX Bone, an alloplastic bone graft substitute (OraPharma Inc., Warminster, PA, USA), and recombinant human bone morphogenetic protein‐2 (rhBMP‐2). The grafted area was then covered with OSSIX Agile, a resorbable, cross‐linked collagen membrane (OraPharma Inc., Warminster, PA, USA) to support guided bone regeneration. Tension‐free primary closure of the surgical site was achieved (Figure 2B–D).

**FIGURE 3:**
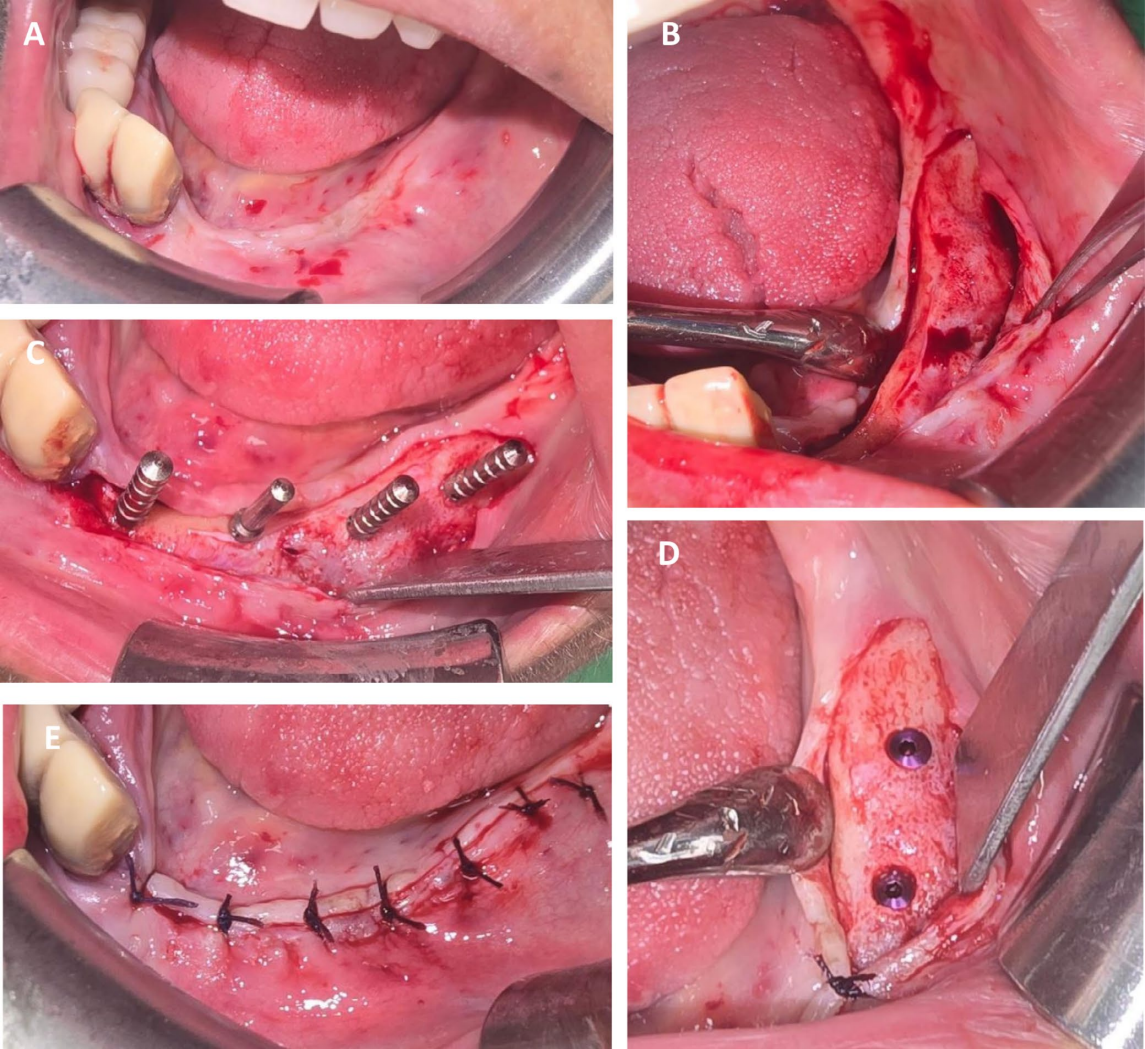
Dental implantation procedure 6 months after bone augmentation. (A) Preoperative intraoral view showing fully healed and healthy soft tissue. (B) Intraoperative exposure of well‐integrated augmented bone. (C) Parallel placement of four dental implants in the left mandible. (D) Final positioning of the dental implants. (E) Primary closure of the surgical site.

The dental implantation procedure was performed 6 months following successful bone augmentation, once complete soft tissue healing and bone regeneration were confirmed (Figure 2E). Intraoperative findings revealed healthy, well‐integrated augmented bone, providing a stable foundation for implant placement. Four dental implants were inserted in the left mandible (Figure 3).

### 2.2 Case 2

#### 2.2.1 Case History and Examination

**FIGURE 4:**
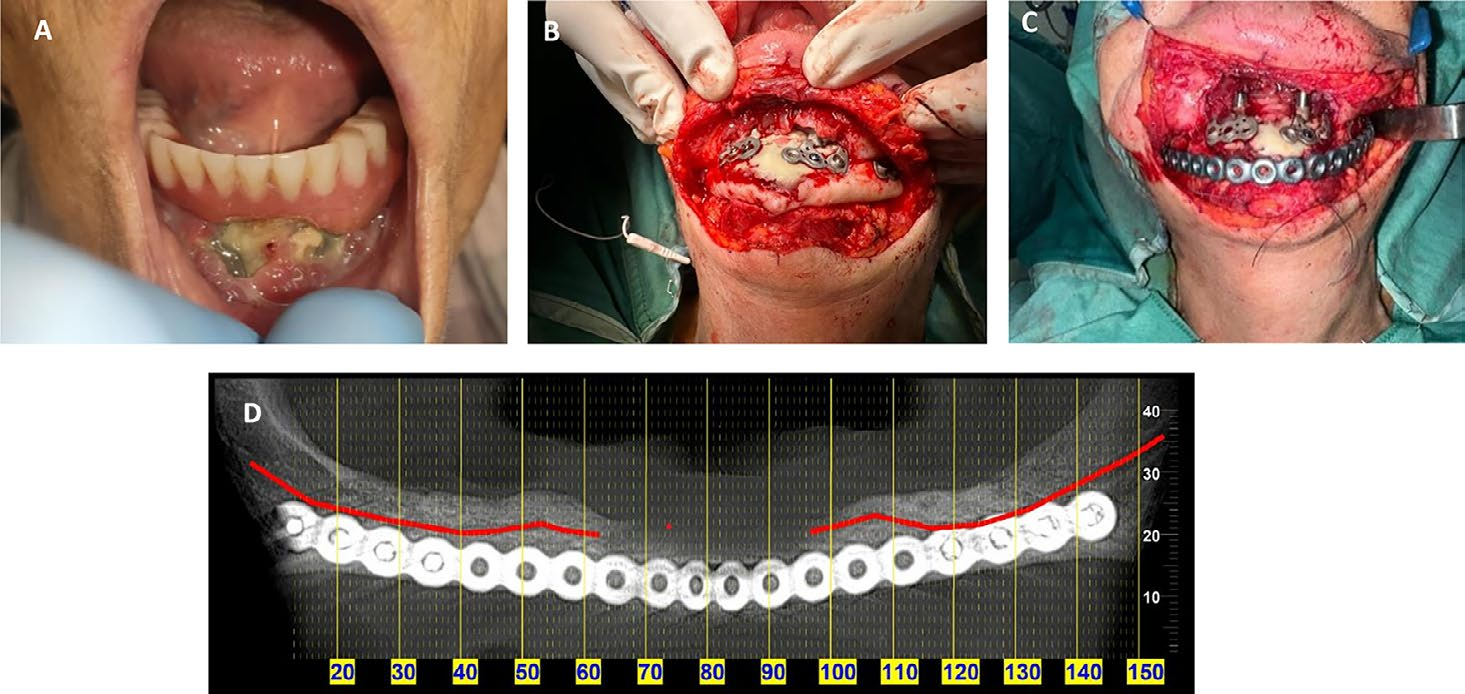
Case history, examination and surgical approach for marginal mandibulectomy—Case 2. (A) Intraoral clinical photograph at initial presentation showing purulent discharge from an intraoral fistula in the anterior mandible. (B) Intraoperative view demonstrating exposure of necrotic mandibular bone and the presence of a failing patient‐specific implant (PSI). (C) Intraoperative image illustrating adaptation and positioning of a reconstruction plate prior to resection of the necrotic bone. (D) Panoramic reconstruction of CBCT of post marginal mandibulectomy and reconstruction plate fixation.

A 74‐year‐old female (D.V.) presented with a sudden onset of pain and swelling in the anterior mandible. Clinical examination revealed an exposed bone with purulent discharge from both an intraoral fistula and an extraoral submental fistula. The patient's medical history included osteoporosis, for which she had been receiving monthly intravenous denosumab for over 3 years. Additionally, she had a past history of ovarian cancer, treated surgically with resection of the ovaries and hysterectomy combined with adjuvant chemotherapy, and was under treatment for hyperlipidemia with Atorvastatin. Three months prior to presentation, she underwent multiple tooth extractions followed by placement of patient‐specific implants (PSI) in the anterior mandible. A CBCT scan at the first visit showed clear evidence of an osteolytic lesion in the anterior mandibular region consistent with stage 3 MRONJ (Figure 4).

#### 2.2.2 Methods (Differential Diagnosis, Investigation, and Treatment)

The initial treatment involved a 2‐month course of antibiotic therapy and a daily chlorhexidine mouth rinse, combined with a discontinuation of Denosumab therapy. The patient underwent marginal resection of the necrotic anterior mandibular bone and removal of the PSI, all under general anesthesia. A reconstruction plate was placed during the same procedure to maintain mandibular continuity (Figure 4), histopathological examination approved MRONJ diagnosis.

**FIGURE 5:**
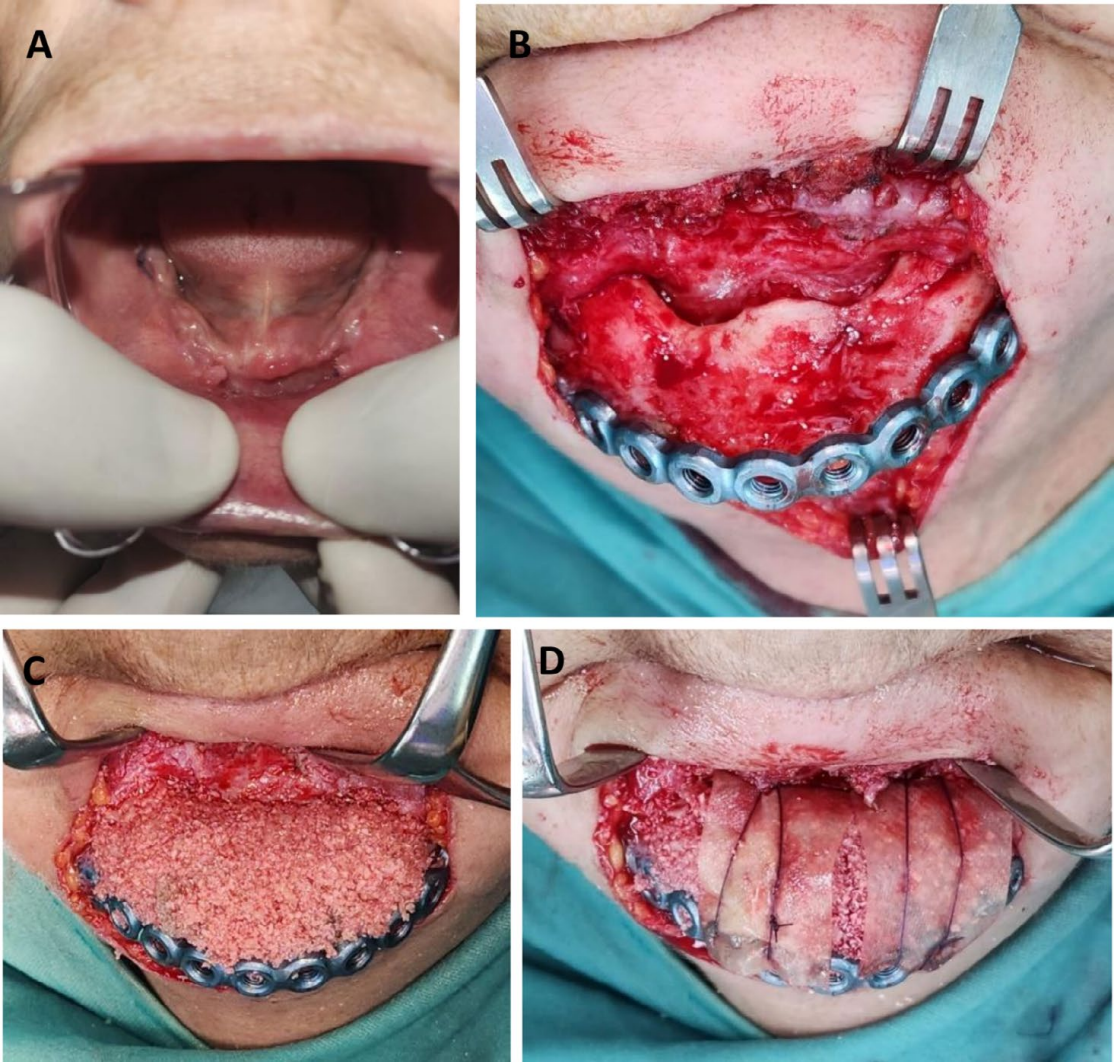
Treatment outcome and follow‐up—Case 2. (A) Intraoral clinical photograph showing complete soft tissue healing in the anterior mandible 3 months following marginal resection. (B–D) Intraoperative images demonstrating the extraoral surgical approach for bone augmentation, utilizing an autologous graft harvested from the anterior iliac crest in combination with recombinant human bone morphogenetic protein‐2 (rhBMP‐2), BIO‐OSS allograft, and OSSIX Agile resorbable membrane.

**FIGURE 6:**
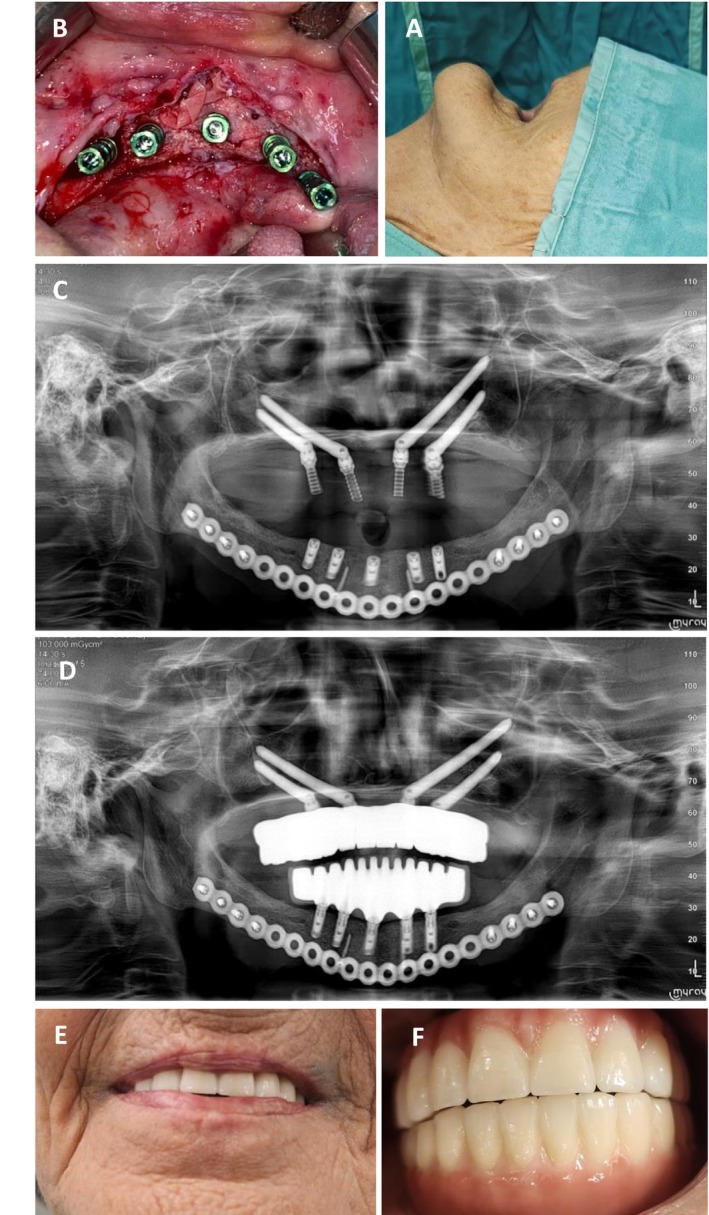
Complete implant‐supported reconstruction—Case 2. (A) Preoperative profile view showing the ill lip support by the atrophic jaws. (B) Intraoperative mandibular dental implantation procedure. (C) 1 week follow‐up panoramic radiograph demonstrating the positioning of the dental implants in the mandible and the zygomatic implants. (D) Panoramic radiograph demonstrating the final prosthodontic treatment. (E, F) Intraoral view of the final prosthodontic treatment demonstrating the functional and esthetic rehabilitation following complete implant‐supported reconstruction.

Three months later, a secondary surgical procedure was performed for bone augmentation. The mandibular defect was reconstructed using an autologous bone graft harvested from the anterior iliac crest, in combination with BIO‐OSS Bone, a xenogeneic bone substitute (Geistlich Pharma AG, Wolhusen, Switzerland), and recombinant human bone morphogenetic protein‐2 (rhBMP‐2). The grafted area was then covered with OSSIX Agile, a resorbable, cross‐linked collagen membrane (OraPharma Inc., Warminster, PA, USA) to support guided bone regeneration (Figure 5). Five months afterward, a complete two‐jaw implant‐supported rehabilitation had been done using five dental implants in the lower jaw and four zygomatic implants (Figure 6).

### 2.3 Case 3

#### 2.3.1 Case History and Examination

**FIGURE 7:**
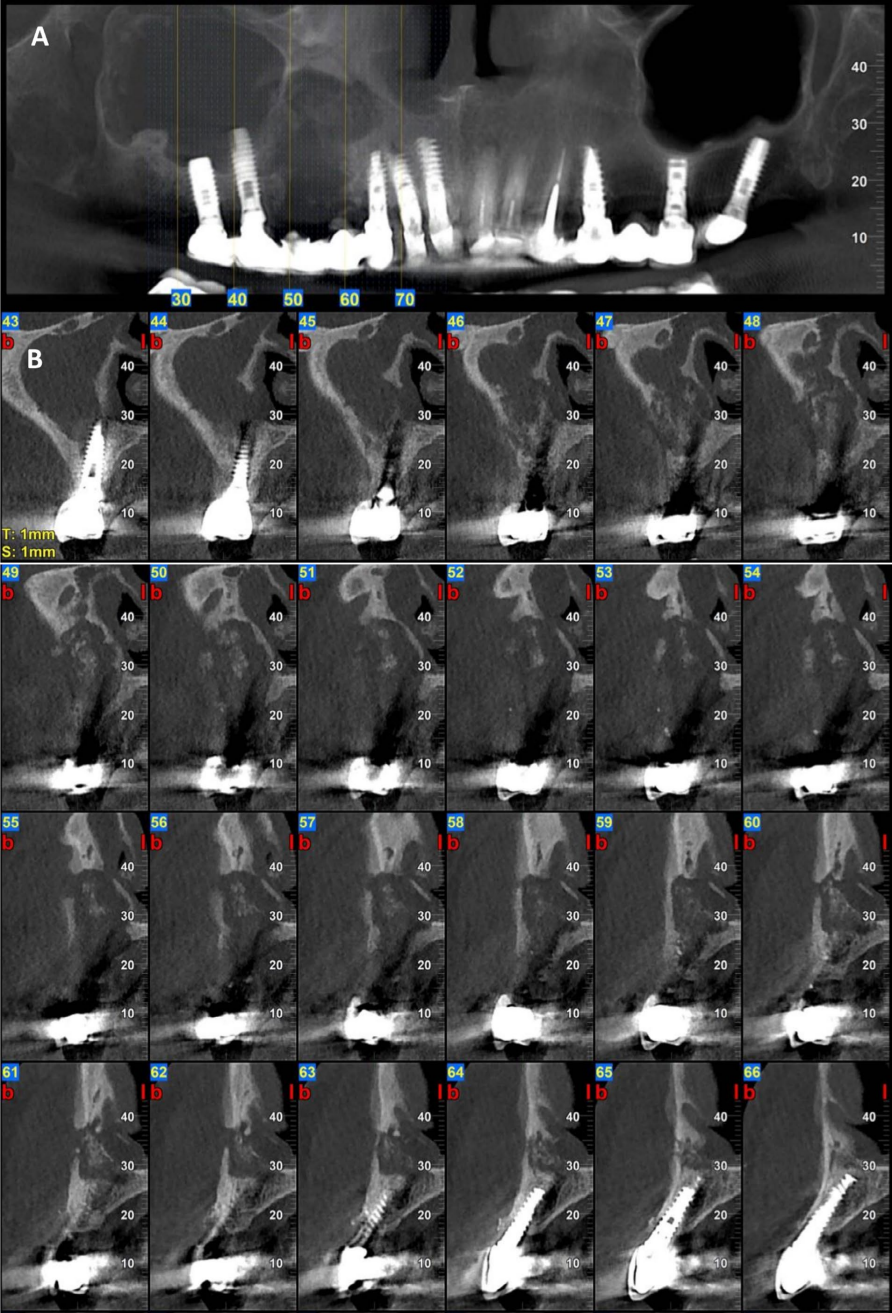
Cone‐beam CT imaging at initial presentation—Case 3. (A) Panoramic reconstruction from cone‐beam computed tomography (CBCT) reveals a well‐defined osteolytic lesion involving the right posterior maxilla, extending between dental implants and into the maxillary sinus. (B) Axial CBCT view demonstrating pronounced osteolysis with visible sequestrum.

A 61‐year‐old woman (T.R.) presented to our clinic with severe pain and noticeable swelling in the right posterior maxilla. Clinical examination revealed a persistent intraoral fistula with purulent discharge and exposed necrotic bone, easily identified upon probing. Radiographic evaluation via cone‐beam CT (CBCT) demonstrated a large osteolytic lesion with clear signs of sequestration and complete maxillary sinus opacification consistent with MRONJ stage 3 (Figure 7).

**FIGURE 8:**
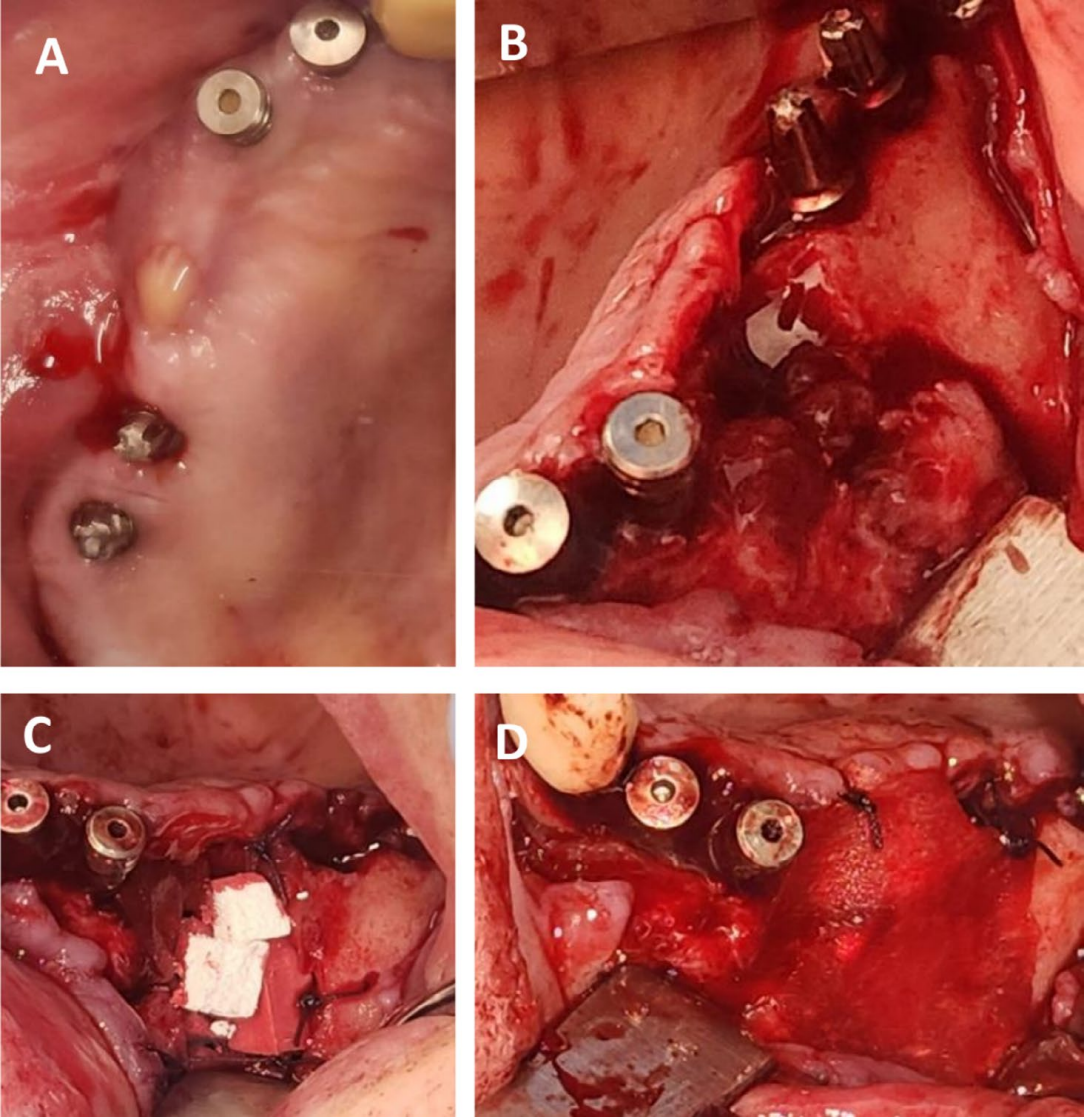
Marginal maxillectomy and bone augmentation—Case 3. (A) Preoperative intraoral clinical view showing purulent discharge from the right maxilla. (B) Surgical reveal of the necrotic area revealing a bone destruction area. (C) Bone augmentation of the defect using OSSIX Bone, a collagen‐based alloplastic bone graft substitute. (D) The graft was covered with OSSIX Agile membrane.

The patient's medical history is significant for osteoporosis, managed with oral bisphosphonates (Alendronate) for over 4 years. Notably, she also suffers from diabetes mellitus, for which she is prescribed metformin, and asthma, managed with inhaled corticosteroids. About 3 months prior to presentation, she reported increasing pain and purulent drainage around dental implants placed in the affected area; prior to that she underwent a failing dental implant removal. Given the local clinical signs, a diagnosis of stage 3 MRONJ was made, and the patient was referred for surgical management (Figure 8).

#### 2.3.2 Methods (Differential Diagnosis, Investigation, and Treatment)

Initial management involved a conservative approach with systemic antibiotic therapy, antimicrobial mouth rinses, and a temporary discontinuation of bisphosphonate therapy (Alendronate).

The patient underwent sequestrectomy and peripheral ostectomy (partial maxillectomy) under local anesthesia. A biopsy was obtained during the same procedure, and histopathological analysis confirmed the diagnosis of MRONJ. Simultaneously, GBR was performed using OSSIX Bone, a collagen‐based alloplastic bone graft substitute (OraPharma Inc., Warminster, PA, USA). The graft was covered with two OSSIX Agile membranes, resorbable cross‐linked collagen barriers (OraPharma Inc., Warminster, PA, USA). Tension‐free primary closure of the soft tissue was achieved (Figure 8).

**FIGURE 9:**
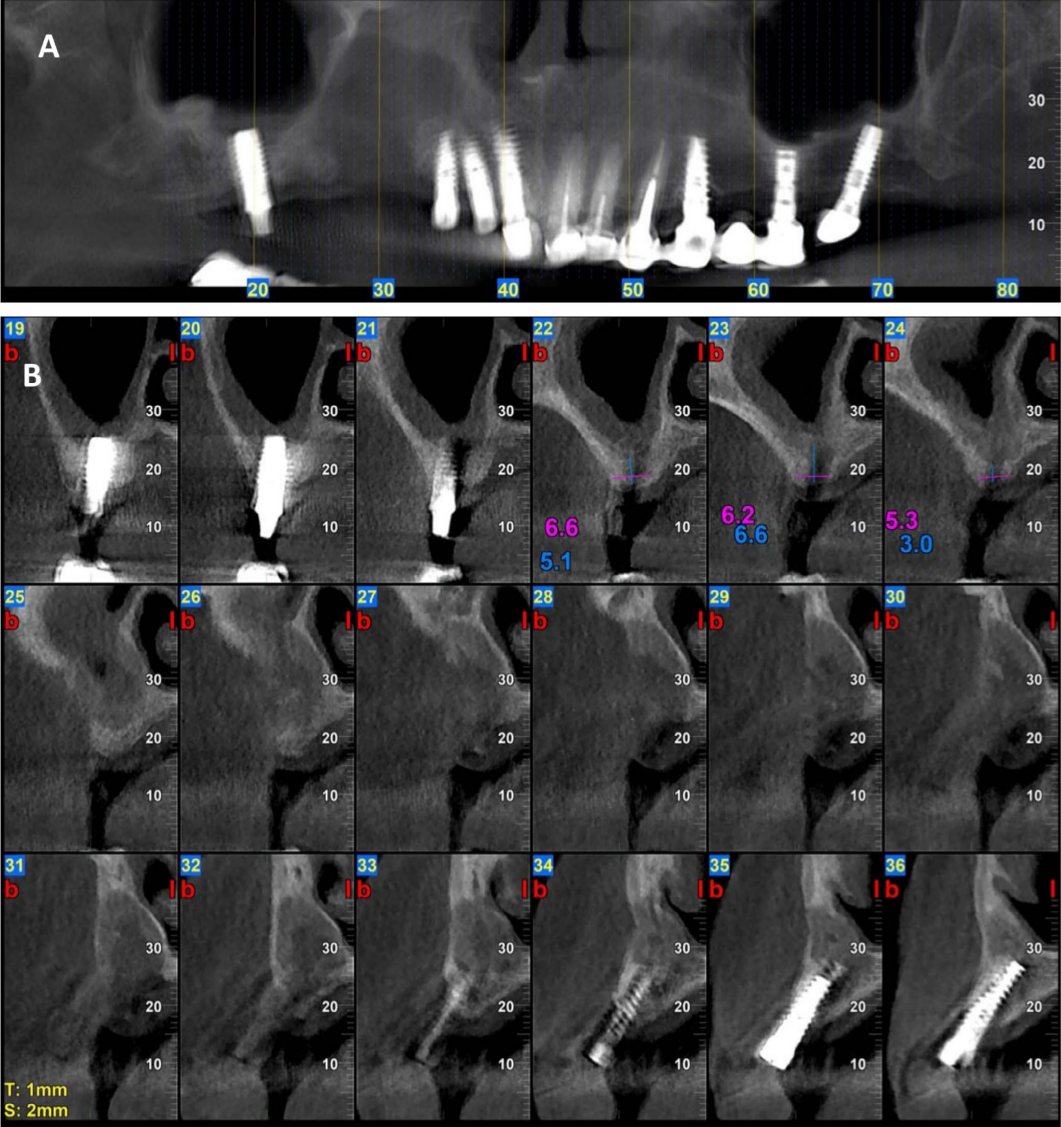
CBCT 4 months follow‐up CBCT post marginal maxillectomy and GBR—Case 3. (A) Panoramic reconstruction from CBCT 4 months after dental implant extraction, marginal maxillectomy of the osteolytic lesion and GBR. (B) Axial CBCT view demonstrating the maxillary defect.

**FIGURE 10:**
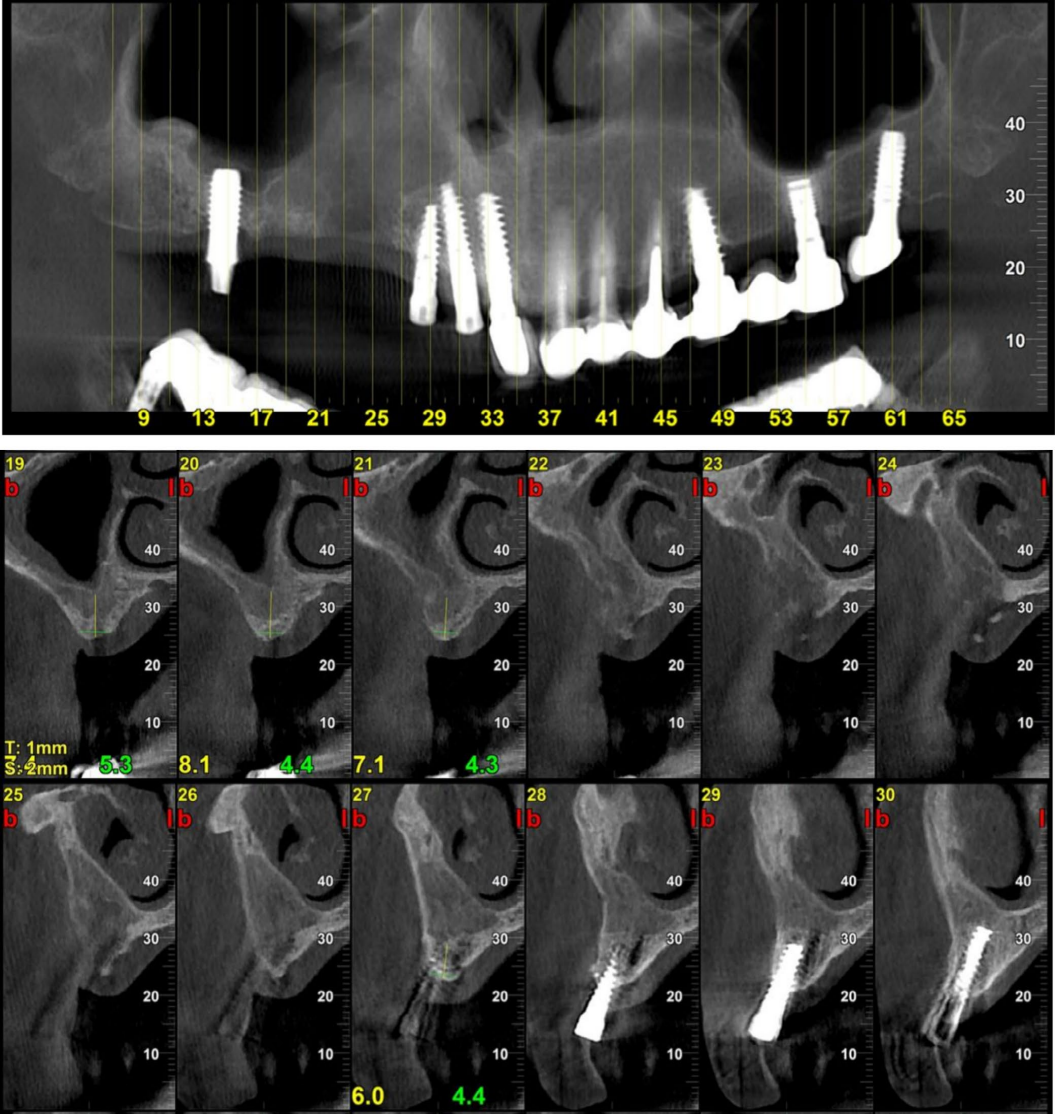
CBCT imaging at 9 months follow‐up post marginal maxillectomy and GBR—Case 3. Panoramic reconstruction from CBCT and axial views reveals a new bone formation on the right posterior maxilla filling the residual space.

The patient has since returned for regular follow‐up visits every 3 months. Clinical evaluations demonstrate progressive soft tissue healing, and serial radiographs reveal encouraging signs of bone regeneration in the affected maxillary region (Figures 9 and 10).

## 3 Conclusion and Results (Outcome and Follow‐Up)

### 3.1 Case 1

The patient attended monthly follow‐ups, which demonstrated favorable healing both intra‐orally and extra‐orally. Radiographic imaging, including CBCT and panoramic X‐rays, confirmed progressive bone regeneration in the treated site (Figure 2E). At 9 months post‐augmentation, dental implants were successfully placed in the previously reconstructed area (Figure 3). The patient remained free of symptoms, with no signs of recurrent infection or necrosis observed during follow‐up visits. Additionally, the patient regained full oral function and reported a significant improvement in quality of life.

### 3.2 Case 2

Follow‐up examinations at 1 and 3 months post‐resection showed complete resolution of inflammation and full soft tissue healing. A CBCT scan performed 3 months after the augmentation procedure revealed substantial bone regeneration with a vertical height of 20–22 mm in the anterior mandible.

Four months following bone augmentation, dental implants were successfully placed in the mandible, and zygomatic implants were inserted in the maxilla to support fixed full‐arch prostheses (Figure 6). The patient remained asymptomatic throughout the postoperative period. The treatment objectives were achieved, including restoration of mandibular continuity and reestablishment of normal oral function, encompassing mastication, speech, and satisfactory esthetics.

### 3.3 Case 3

At the 1‐month follow‐up, clinical examination demonstrated normal soft tissue healing and evidence of healthy bone formation in the treated region. The patient reported complete resolution of symptoms, with restoration of a healthy and functional maxillary sinus. No signs of recurrent infection or oroantral fistula were observed. Radiographic imaging confirmed early signs of successful bone regeneration. Ongoing follow‐up is planned to assess long‐term stability, with future treatment including the placement of dental implants and full restoration of oral function (Figures 8 and 9).

## 4 Discussion

This case series illustrates three examples of MRONJ in osteoporotic patients treated with antiresorptive medications. Despite differences in anatomical location, antiresorptive agent, and surgical details, all three cases shared common features: a history of dentoalveolar procedures without prior drug holiday, clinical presentation of exposed or probed necrotic bone with purulent discharge, and radiographic evidence of osteolysis. Each diagnosis was confirmed by a histopathological evaluation. All patients were successfully treated using a combined approach of surgical resection and GBR, which not only avoided the need for extensive reconstructive procedures in medically compromised individuals but also led to significant improvements in quality of life—restoring masticatory function, speech, and facial esthetics through the rehabilitation of a functional dentition.

Consistent with the literature, the most prominent local risk factor observed in these cases was recent dentoalveolar surgery (implant removal or tooth extraction) performed without the suspension of antiresorptive therapy. This aligns with data indicating that dentoalveolar procedures precede up to 82% of MRONJ cases [10, 11]. Furthermore, the chronic use of antiresorptive agents in these patients (ranging from 4 to over 7 years) contributed to the cumulative systemic risk [4].

Despite the documented reluctance among clinicians to perform surgical intervention in MRONJ patients due to the potential of exacerbating the disease [4], our experience reinforces a growing body of evidence supporting surgical management as a definitive treatment modality. Each case underwent marginal resection (mandibular or maxillary) and showed complete resolution of infection and fistulae, without postoperative complications. The success of these interventions corresponds with findings from recent studies that report surgical treatment achieving significantly higher healing rates compared to conservative management [4]. These cases add to the evidence that surgical intervention can play a therapeutic role when performed in a controlled, protocol‐driven manner.

Moreover, our approach prioritized surgical resection of the necrotic lesion followed by immediate or delayed bone regeneration using a combination of autogenous grafts, bone substitutes, membranes, and rhBMP‐2. This aligns with our institutional goal to minimize the morbidity associated with major reconstructive surgeries involving free flaps or large vascularized bone segments. Instead of pursuing radical procedures with higher complication risks, we adopted a biologically favorable environment for healing through GBR, even in compromised sites with prior infection. In all three patients, follow‐up imaging confirmed bone regeneration, and two of them successfully received dental implants within the previously necrotic areas—underscoring the functional and structural rehabilitation potential of this technique.

In conclusion, the outcomes observed in this series support the evolving perspective that surgical resection, when coupled with GBR and appropriate medical management, can offer a safe and effective solution for MRONJ in selected patients. This reinforces the need to reevaluate conventional hesitations regarding surgery in MRONJ and instead advocate for tailored, evidence‐informed treatment protocols that aim to preserve function and improve patient quality of life.

## Author Contributions

**Daya Masri:** conceptualization, data curation, formal analysis, investigation, methodology, project administration, supervision, validation, visualization, writing – original draft, writing – review and editing. **Omar Ghanaiem:** conceptualization, data curation, formal analysis, investigation, methodology, project administration, supervision, validation, visualization, writing – original draft. **Vadim Raiser:** investigation, methodology, supervision, visualization. **Shaked Adut:** investigation, methodology, project administration, supervision. **Gavriel Chaushu:** conceptualization, investigation, methodology, supervision, validation, visualization.

## Consent

Written informed consent was obtained from parents to publish this case report in accordance with the journal's patient consent policy.

## Conflicts of Interest

The authors declare no conflicts of interest.

## Data Availability

The data that support the findings of this study are available on request from the corresponding author. The data are not publicly available due to privacy or ethical restrictions.
